# The American Dental Association

**Published:** 1869-09

**Authors:** W. C. Horne

**Affiliations:** New York.


### ARTICLE IV.

### *The American Dental Association.*

By W. C. Horne, D.D.S., New York.

The ninth annual meeting of the American Dental Association was held at Saratoga Springs, New York, commencing on Tuesday, August 3, 1869. There was an attendance of one hundred and thirty-six members.

The Association was called to order at 11 o'clock by the President, Dr. Jonathan Taft, and the session opened with prayer by the Rev. John Woodbridge, D.D.

Dr. J. G. Ambler, of New York, Chairman of the Committee of Arrangements, delivered the usual address of welcome; which was followed by the roll-call.

The Report of the Committee on Dental Pathology and Surgery was presented and read by Dr. Atkinson.

The hours of business were then appointed, and an adjournment taken to 3 o'clock. The whole of the afternoon session was occupied with discussions upon Dental Pathology and Surgery.

SECOND DAY.

The Treasurer presented his report, which was referred to an auditing committee ; and the discussion on Dental Pathology and Surgery was resumed.

The Committee on Dental Chemistry failing to report, Dr. T. L. Buckingham made, by request, a verbal report.

The rules were now suspended to allow Professor Trueman to offer two resolutions: one directing the Treasurer to refund certain dues claimed to have been illegally demanded; and the other recommending dental societies to admit female practitioners to membership. The resolutions were temporarily laid on the table.

The discussion of Dental Chemistry ensued ; after which the time of final adjournment was fixed at 5 o'clock of Friday.

At the opening of the afternoon session Dr. C. R. Butler presented the report of the Committee on Operative Dentistry. The rules were then suspended, and the following Nominating Committee was appointed, and instructed, for the present, to nominate the standing committees only.

W. W. Allport, C. Francis, M. S. Dean, T. L. Buckingham, Homer Judd, L. D. Shepard, A. H. Brockway, A. L. Northrop, C. W. Robinson.

The regular order being resumed, Dr. C. Palmer made an additional report on Operative Dentistry, illustrated by large diagrams and models of the superior and inferior dental arches ; and Dr. Perkins presented a patient who had lost the entire inferior maxilla from phosphor-necrosis.

The Auditing Committee, consisting of Drs. M. S. Dean, E. A. Bogue, and L. D. Shepard, to whom the Treasurer's account was referred, reported it to be correct. They expressed the opinion that the permanent members consist of all those who have once attended as delegates, and that such persons remain permanent members until, their dues being paid in full they voluntarily withdraw, or are dishonorably dropped from the rolls for non-payment of dues. They also recommended the adoption of the following resolutions:

*Resolved*, That a dentist having once appeared as a delegate, and become a permanent member, is not eligible to act again as a delegate until his dues are paid in full.

After a sharp debate this resolution, on a call of the yeas and nays, was adopted by a vote of 29 to 28; the President voting in the affirmative.

By permission, Dr. Horne changed his vote to the affirmative; after which he moved a reconsideration, which was rejected.

THIRD DAY.

Dr. H. Judd presented the report of the Publication Committee, which showed a balance of $152.78 to be due them. The Committee published five hundred copies of the Transactions for 1868, at a cost of $475. The report was accepted, and the Committee discharged, with the thanks of the Association, and the balance due ordered paid.

The Nominating Committee reported the names of Standing Committees for the ensuing year. The report was recommitted, with instructions to make certain changes, and to nominate officers.

Dr. Atkinson offered a resolution to refer to the Committee on Dental Literature a new work of Dr. J. E. Garretson, entitled "Diseases and Surgery of the Mouth," which he commended very highly, as the last and most accurate statement of the condition of medical knowledge in this department. The Committee declined to consider the subject, from lack of time, and the resolution was laid on the table.

The Committee on Prize Essays made the usual report, that nothing had been presented for their consideration.

Discussion upon Operative Dentistry was then commenced, and occupied the rest of the morning session.

At the commencement of the afternoon session, after much ballotting, the City of Nashville was selected as the next place of meeting.

Dr. Morgan said he wanted every member of the Association to feel that he was bound to be present at the next meeting in Nashville. He related of Professor Agassiz, that on being requested to visit various cities to lecture, he replied that he had not time to be running about making money, he had more important business to attend to. He (Dr. M.) desired members to feel that it was of more importance to them to attend the annual meetings than to stay at home and make money.

Dr. Atkinson said he had been requested by Dr. Evans, of Paris, to say that he had expected to be present at this meeting (having been mistaken as to the date of its session), but that he had to return to Paris to be present on the fete day of his pet emperor. He had been greatly pleased with what he saw of Dr. Evans, during his short stay; he was one of the few men who could be petted without being spoiled; he had received without solicitation, many orders of knighthood; and he (Dr. A.) indorsed him as a Christian and scholar, Though dwelling so long in a foreign land, he had maintained his loyalty to American principles and American dentistry, and he desired to be so recognized by his fellows in this Association.

The Committee on Nominations then made the following report:

FOR OFFICERS.

*President.*—Homer Judd, St. Louis; W. W. Allport, Chicago.

*First Vice-President.*—S.J. Cobb,Nashville; J.F. Knapp, New Orleans.

*Second Vice-President.*—C.E.Francis, New York; W.H. Shadoan, Louisville.

*Corresponding Secretary*—I. A. Salmon, Boston; H. J. Smith, Illinois.

*Recording Secretary.*—W. C. Horne, New York; M. S. Dean, Chicago.

*Treasurer.*—W. H. Godard, Louisville.

STANDING COMMITTEES.

*Committee of Arrangements.*—W. H. Morgan, S. J. Cobb, W. H. Shadoan,

*Committee on Publication.*—M. S. Dean, E. A. Bogue, J. Taft.

*Committee on Prize Essays.*—G. T. Moffatt, J.F. Adams, H. G. Mirick, S. M. Cummings.

*Committee on Dental Physiology.*—J. H. McQuillan, Jas. Truman, H. F. Bishop.

*Committee on Dental Chemistry.*—T. L. Buckingham, John Allen, G. R. Thomas.

*Committee on Dental Pathology and Surgery.*—W. H. Atkinson, J. S. Knapp, C. R. Butler.

*Committee on Operative Dentistry.*—J. Taft, George H. Cushing, Corydon Palmer.

*Committee on Mechanical Dentistry.*—W. H. Eames, S. B. Palmer, Z. Cotton, L. M. Sturgis.

*Committee on Dental Literature.*—L. D. Shepard, J. Mc Manus, H. J. Smith.

*Committee on Vountary Essays.*—I. J. Wetherbee, C. D. Cook, L. S. Straw.

*Committee on Dental Histology.*—Homer Judd, W. W. Allport, R. W. Varney.

*Committee on Dental Therapeutics.*—T. B. Hitchcock, C. N. Pierce, G. F. Waters.

*Committee on Dental Instruments and Appliances.*—Frank Abbott, A. H. Holmes, J. B. Morrison.

The Standing Committees were confirmed. An evening meeeting was then ordered to receive the report of the Committee on Amendments to the Constitution.

At 8 o'clock the evening session was opened, and the above named report read and accepted. After various motions to adopt, to recommit, etc., the whole subject was laid on the table.

An election of officers was then held.

Drs. Judd, Morgan, and Allport were voted for, and, after several ballots, Dr. Homer Judd was elected President; Dr. S. J. Cobb and Dr. C. E. Francis, Vice-Presidents; Dr. I. Salmon, Corresponding Secretary; Dr. M. S. Dean, Recording Secretary; Dr. W. H. Goddard, Treasurer.

The Association then adjourned to the next morning.

FOURTH DAY.

A committee of five was ordered to make arrangements for reduction of railway fares to Nashville next year, namely T. L. Buckingham, I. J. Wetherbee, E. A. Bogue, G. H. Cushing, G. R. Thomas.

Dr. McQuillen, Chairman of the Committee on Histology, made a verbal report, accompanied by a number of microscopical specimens recently prepared by him. 1, of injected pulps of calves' teeth ; 2, of the kidney of the sheep ; 3, of the muscles of three persons who had died within the past year of trichiniasis, along with a portion of the pork, containing trichinæ, which had caused the disease of one of the deceased ; after which the subject was discussed.

The report from the Committee on Mechanical Dentistry was presented by Dr. John Allen,who regretted that, while the operative branch of dentistry had advanced so much within a few years, in this department, the general course of dentists, had been to make the cheapest instead of the best work. The difficulty of obviating the discrepancy between the mouth and the dies made from the impression was admitted, but the idea of remedying this by resorting to a plate of lighter material was controverted as false in principle, which was exemplified by the simple experiment of a sheet of paper supported upon the mouth of an inverted tumbler full of water. There is demand, then, for a process, which shall insure mathematical accuracy in the fitting of the plate; as well as great need of skill in the arrangement of teeth to conform with the characteristics of the face.

He was followed by Dr. S. B. Palmer of Syracuse, with an essay on "Repairing Vulcanite," and by Dr. J. A. Mc Clelland with an essay on the "Collodion Base."

The essay of Dr. Palmer is explanatory of a method of thoroughly repairing broken rubber-plates by varnishing the surfaces, to which the new rubber is to be attached, with a creamy solution of rubber in chloroform ; to be kept on hand for such use. He states that repairs made in this way are perfectly reliable, even if the broken edges are only beveled, without dovetailing or perforating the old piece. Wax, gutta-percha, oil, or soap are agents which prevent rubber from being vulcanized, and they should therefore, be carefully kept from contact with any piece to which it is intended to apply this process.

The Essay on "Consolidated Collodion, Pyroxylin, or Rose Pearl," is an enthusiastic description of the method of preparing that material for use in dental plates. It is prophetically characterized as "the coming base." The time required for the evaporation of the ether seems to be an inconvenience, "because we have become so demoralized in our ideas of time by the use of a cheap substance (rubber) that requires but a few hours and little skill to make into plates." "In practice the time required for 'Rose Pearl, to fit *herself* for the mouth is soon regarded as gain rather than loss." The shrinkage of the material is said to be controlled by such simple means that the cry, "It shrinks!" becomes one of ridiculous insignificance to the friends of "Rose Pearl."

Dr. Corydon Palmer exhibited an improved moulding flask, and explained its advantages in difficult cases. A vote of thanks to Dr. Palmer was passed (which the Secretary was instructed to have handsomely engrossed) for the manner in which he had presented, by means of plaster models and diagrams, an advanced method of preparing and filling teeth, and an appropriate classification of fissures where teeth are most liable to decay.

The report of the Committee on Voluntary Essays was presented and adopted.

Dr. M. S. Dean, from the Committee on Dental Education, presented a report on the importance of a thorough preliminary education for dental students, and was followed by Dr. S. B. Palmer, with an essay on "Dental Education for the People."

Dr. Palmer advocated the diffusion of knowledge in regard to the preservation of the dental organs by means of tracts or periodicals. He believed there was great necessity for for such information, and that it would be highly appreciated.

Dr. Cobb indorsed the sentiment of the essayist; he was greatly impressed with the ignorance of educated people in regard to their teeth; all that the community know in regard to such matters is the little they pick up in the dentists' offices. He held it to be the duty of practitioners to instruct their patients. Many more people would have their teeth preserved if they knew that it was true economy to do so. He strongly commended the plan of the *People's Dental Journal*, and was much in favor of the distribution of tracts to increase popular dental knowledge. There would be vastly more dental work done if the people knew the importance of it; something in the form of a catechism, or instructions which might be introduced into schools, was a *desideratum*. No branch of knowledge was more neglected, and none would insure more immediate good results by its propogation. It was a common idea that the charges of dentists were exorbitant, whereas they were far more moderate in proportion than those of physicians and general surgeons.

Dr. McDonald advocated the preparation of tracts, under the auspices of the Association, for distribution among the people. Early instruction in regard to the value of the teeth, and proper means of caring for them, would be of immense value to the American people and to American dentists. A great many more teeth would be filled, but there would eventually be a great many less large operations to be performed, and, consequently, a great deal better condition of the teeth might be insured at much less expenditure of money.

The Committee on Dental Literature had no report.

The Committee on Dental Therapeutics made a very brief report by Dr. Bogue.

The report of the Commmittee on Dental Instruments and Appliances was presented Drs. F. Abbott and C. Palmer. They noticed improvements in dental chairs by J. B. Morrisson and O. C. White; a plating for instruments of pure nickel, by M. M. Johnson; an imstrument for rolling gold foil, by J. B. Adams, of Worcester; an instrument for regulating heat in the manufacture of nitrous oxide, where ordinary burning gas is used, by J. P. Collidge, of Boston; clamps and buttons to close the duct of Steno, by B. T. Whitney; pneumatic mallet by W. H. Jackson, of Ann Arbor; an improved regulator and heater where kerosene is used in making nitrous oxide, by A. W. Sprague; burs by S. S. White, of finest steel regularly divided and evenly cut, which instead of being left with the file finish as in ordinary burs, are, after hardening, finished with a stone to an edge as fine as a lance-blade, so that in the hands of a sufficiently skillfull operator they will cut with the least possible pressure, avoiding almost entirely the unpleasant sensation of ordinary burs; artificial teeth, by S. S. White, which the Committee stated were the finest they had seen, in their expression, and proportion between the upper and lower sets; nitrous oxide, ether, and chloroform inhalers, by Dr. Wilson, securing greater safety in the use of these inhalers by insuring perfect control of the supply of atmospheric air, in well-defined proportions.

The report of the Executive Committee was then presented and adopted.

The report of the Committee on Amendments to the Constitution was taken from the table, and the report was adopted without even a reading of it.

Dr. Truman's resolution on the right of female dentists to membership was indefinitely postponed, because the Association had no right to make recommendations to local societies.

Dr. Buckingham gave notice of an amendment to the Constitution, to be acted upon next year, providing that no person who holds a dental patent, or is pecuniarily interested therein, shall be a member of the Association.

Dr. W. H. Shadoan offered a resolution donating the amount of back dues, from 1865, to 1869, to thirty-three members, who were reported by him to be arrears, each to the amount of $23. The resolution passed after an animated debate.

The Committee on Ethics reported, through Dr. Shepard, that they had had brought before them charges against Dr. J. A. McClelland, of Louisville, for violating Article II., Section 3, of the Code of Ethics, by placarding large advertisements on the street cars of Louisville, and by unprofessional advertisements in the papers, which were read ; they therefore offered the following resolution : "That J. A. McClelland, of Louisville, be expelled from this Association."

They also reported that they found upon the records of the Association charges against Dr. C. P. Fitch, of New York, for violation of the same clause of the Code of Ethics ; but they did not feel authorized to recommend action on his case, as no definite charges or proofs had been offered.

Dr. Atkinson called to mind the remark of Dr. McQuillen at the time of the adoption of the Code of Ethics, that it was unnecessary for gentlemen, and useless for those who were not such. He did not like the idea of singling out one or two as examples and leaving all the others to go free. It was well known that Dr. Watt, who had so persistently urged the adoption of this code, had gone home and signally violated its provisions, and yet no one had lifted up a voice against him. He thought the adoption of laws of this nature peculiarly unfortunate ; because they would be brought to bear unequally ; while one would be made to suffer the utmost penalty, others wonld be allowed to go free.

Dr. Fitch asked to be heard in explanation. He said that many loose and unfounded charges were floating about against him. The sum of his offence, he said, was this : that he had advertised the public of New York in good faith that he was ready to operate at reduced prices on certain days and hours ; because there was a large class of most worthy people in that city who were desirous to preserve their teeth, and could not afford to pay the current rates of first-class operators. He had done nothing to lower the standard of professional skill, but only made use of the circumstances of the case to minister to his necessities. He yielded to no man in his love for the profession, and his desire for its advancement. He had meant to do no wrong in any course he had pursued, and, whatever the action of the Association, should endeavor to maintain the character of his professional operations, and devote his efforts to the relief of humanity within the range of his practice.

On a motion being made to refer Dr. McClelland's case to the Committee on Ethics for the ensuing year—

Dr. McQuillen opposed very strongly the postponement, and was in favor of proceeding at once with the trial of this case, which was a most flagrant one. As already stated, he had objected to the adoption of a code of Ethics; but since it had become part of the organic law of the Association, he demanded its enforcement. While it was mortifying to know that the Code had been violated by one who had prepared it, and was most zealous in forcing it upon the organization, yet it was not an unusual thing in the history of morals for men to make laws and then to be the first to break them. It was much better to make few if any professions, and rather exceed than fall short of such as are made. We could, however, only deal with cases in which specific and thoroughly substantiated charges had been brought before the Association: two such were under consideration. One of these, Dr. Fitch, had abandoned the objectionable practice, and offered an explanation with the desire of making some reparation; but in the other instance the accused was openly, and in the most objectionable manner possible, pursuing his unprofessional course. The rules of the Association had been so often suspended that there could be no possible objection to doing so then, and proceeding with the trial. The person charged with the offense was present, and no injustice would be done to him, as the members would listen patiently to what he might say in defense of his course before taking action upon it. If there was one class of men in particular for whom he entertained the most profound feeling of pity (he would not say contempt, for one should endeavor to unlearn that) it was those who were so lost to all sense of propriety and decency that they could stoop to the low tricks of charlatans, and thus engage in practices which cast stigma upon themselves and the profession they dishonor. If such as these were to be present as meet companions, it would soon make not only the Association but the profession a by-word and a reproach. What they could want in the organization was difficult to conceive, for they were not with it in spirit, and should not be of it in person. Laws promptly and justly enforced in such a case would exercise a beneficial influence upon the *morale* of the profession.

Dr. Horne stated that the clause under which Dr. McClelland was indicted required that the charges should be investigated and reported upon at the next annual meeting after that at which they were made. The Association had adopted the report of a committee which proposed to substitute a new Constitution without a word of debate. If the old Constitution were in force, Dr. McClelland had the right to a copy of all the charges and specifications, and a year to answer in; if the new one were in force, there was no provision by which he could be brought to trial.

The portion of the report in regard to Dr. Fitch was then adopted; that relating to Dr. McClelland was referred to the Committee on Ethics for the ensuing year. Drs. W. H. Morgan, C. R. Cutler., and L. D. Shepard were appointed as that committee.

A resolution of Dr. Bogue's, expressing regrets at the existence of misapprehension as to certain members (unnamed), and for the injustice of an *ex post facto* interpretation of laws, was laid on the table; and another, by the same, calling for a vote of censure on Dr. Atkinson, for disregarding the rules of order, was replied to by Dr. Atkinson in a characteristic manner. The resolution was ordered to be expunged.

The Publication Committee was instructed to print the Constitution with the Transactions.

Dr. Homer Judd was then inducted as President, and Dr. Taft read an address, after which the Association adjourned to the first Tuesday of August, 1870.

### EDITORIAL DEPARTMENT.

